# Helicobacter pylori infection might be responsible for the interconnection between type 1 diabetes and autoimmune thyroiditis

**DOI:** 10.1186/1758-5996-3-28

**Published:** 2011-10-26

**Authors:** Mervat M El-Eshmawy, Amany K El-Hawary, Soma S Abdel Gawad, Azza A El-Baiomy

**Affiliations:** 1 Internal Medicine Department, Mansoura Specialized Medical Hospital, Faculty of Medicine, Mansoura University, Egypt; 2 Pediatric Department, Mansoura pediatric Hospital, Faculty of Medicine, Mansoura University, Egypt; 3 Clinical Pathology Department, Faculty of Medicine, Mansoura University, Egyp

**Keywords:** Helicobacter pylori, type1 diabetes mellitus, autoimmune thyroiditis

## Abstract

**Background:**

Higher serological prevalence rates of helicobacter pylori (H. pylori) infection have been reported in patients with type 1 diabetes (T1DM) and autoimmune thyroiditis (AT). Patients with T1DM are at increased risk for developing other autoimmune diseases, most commonly AT. It is unknown whether H. pylori infection could explain the high prevalence of thyroid autoantibodies and AT in T1DM. The aim of the current study was to evaluate anti-thyroid peroxidase (anti-TPO) and anti-thyroglobulin (anti-Tg) autoantibodies in correlation with anti-H. pylori IgG and IgA in young patients with T1DM.

**Methods:**

Anti-H. Pylori IgG, IgA, anti-TPO and anti-Tg antibodies titers were measured in 162 euthyroid patients with T1DM and 80 healthy controls matched for age, sex and socioeconomic status.

**Results:**

Seroprevalence of H. pylori was significantly higher in patients with T1DM than in healthy controls; 79% vs. 51.2%, p < 0.001. Anti H. pylori IgG was positive in 61.1% of patients with T1DM and 30% of controls, p < 0.001, anti H. pylori IgA was positive in 74% of patients with T1DM and 32.5% of controls, p < 0.001. Thyroid autoimmunity was also significantly higher in patients with T1DM than in controls; 56.7% vs. 6.2%, p < 0.001. Anti-TPO was positive in 25.3% of patients with T1DM and 3.7% of controls, p < 0.001, anti-Tg was positive in 47.5% of patients with T1DM and 6.2% of controls, p < 0.001. With simple and multiple regression analysis anti-H. pylori IgG and IgA titers were positively and significantly correlated with Anti-TPO and anti-Tg titers in patients with T1DM.

**Conclusion:**

our results support the idea of a connection between H. pylori infection and the occurrence of anti-TPO, anti-Tg autoantibodies and AT in young patients with T1DM. So, H. pylori infection could be considered as an environmental trigger for development of AT in T1DM. Young patients with T1DM should be screened for H. pylori infection.

## Background

Helicobacter pylori (H. pylori) is one of the most common chronic infections worldwide [1,2]. It affects approximately 50% of the world population and more prevalent in developing than in developed countries [3], however, the majority of infected subjects develop no clinical symptoms [4]. H. pylori specifically colonizes the gastric epithelium and causes chronic gastritis, peptic ulcer disease and/or gastric malignancies [5]; moreover, it has been epidemiologically linked to some extradigestive diseases [6]. Higher serological prevalence rates of H. pylori infection have been previously reported in patients with type 1 diabetes (T1DM) [7] and autoimmune thyroiditis (AT) [8].

Patients with T1DM are at increased risk for developing other autoimmune diseases, most commonly AT [9,10]. Up to 20% of patients with T1DM have positive anti-thyroid antibodies; anti-thyroglobulin (anti-Tg) and anti-thyroid peroxidase (anti-TPO) antibodies and 2 to 5% of patients with T1DM develop autoimmune hypothyroidism [11].

Thus, the question arises is whether H. pylori infection could be a reason for the increased prevalence of thyroid autoantibodies and AT in T1DM; so it might be considered as an environmental trigger for development of AT. The aim of the present study was to evaluate anti-TPO and anti-Tg autoantibodies in correlation with anti-H. pylori IgG and IgA in young patients with T1DM.

## Methods

### Selection of patients with T1DM and healthy controls

One hundred and sixty two euthyroid patients with T1DM (90 female and 72 male; mean age: 19.35 ± 2.6 years; diabetes duration: 7.29 ± 7.9) attending out-patient diabetes clinics at Pediatric and Specialized Medical Hospitals, Mansoura University, Egypt were studied (Table 1). The diagnosis and clinical classification of diabetes mellitus were based on the guidelines of the American Diabetes Association [12]. Eighty healthy participants matched for age, sex and socioeconomic status, coming from the same geographic area, were evaluated as controls. A validated questionnaire concerning the presence of dyspeptic symptoms (epigastric pain, bloating, post prandial fullness, nausea and vomiting) was administered. All participants signed an informed consent to be included in our study. This study was approved by the local ethical committee.

**Table 1:** Clinical and Biochemical Parameters of the study subjects

Parameters	Patients with T1DM (n = 162)	controls (n = 80)	*P*- value
Age (years)	19.35 ± 2.6	19.76 ± 2.76	0.3

Females (%)	55.5% (90/162)	55% (44/80)	0.93

Gastrointestinal symptoms	4.3% (7/162)	3.7% (3/80)	0.83

Duration of diabetes (years)	7.29 ± 7.9	_	

HbA1c (%)	8.2 ± 1.75	_	

Data are expressed as mean ± standard deviation, numbers or percentages, T1DM; type 1 diabetes mellitus, HbA1c; glycated hemoglobin.

Exclusion criteria: None of the patients had goiter, known peptic ulcer disease or complications attributable to diabetic vasculopathy. None of the diabetic or control subjects used antibiotics, anti-inflammatory drugs (which may interfere with dyspeptic symptoms), prokinetics or proton pump inhibitors in the 2 months before testing.

### Biochemical and antibodies assay

Free-thyroxine (FT4) and thyroid stimulating hormone (TSH) were measured by electro-chemiluminecent immunoassay, using Elecsys 2010, Roche Diagnostic, Germany. Glycated hemoglobin A1c (HbA1c) was measured as an index of metabolic control on a DCA 2000 analyzer. Fast ion exchange resin supplied by human (Germany). The normal range was 4.4%- 6.4%.

Serum anti-H. pylori antibodies IgG and IgA were assayed by sequential ELISA method supplied by Monobind Inc (USA). Results were considered positive when the titers were higher than 20 u/ml. Anti-TPO and anti-Tg autoantibodies were estimated by immunoenzymatic assay supplied by Diametra Italy. Results were considered positive when the titers were higher than 20 Au/ml and 4 Au/ml respectively.

Patients with T1DM and healthy control were considered to be H. pylori positive when anti-H. pylori IgG and/or IgA were positive and showing signs of thyroid autoimmunity when anti-TPO and/or anti-Tg were positive.

### Statistical analysis

Data entry and analyses were performed using SPSS statistical package version 10 (SPSS, Inc., Chicago, IL, USA). The quantitative data were presented as a mean and standard deviation and the qualitative data were presented as number and percentage. The chi-square (χ^2^) was used to find the association between qualitative data. Student's t-test was conducted to compare the mean of continuous variable in two groups. Odds ratio was calculated to determine risk of occurrence with 95% confidence interval. Simple correlation and multiple regression analysis of anti-H. pylori IgG and IgA antibodies titers with other parameters in patients with T1DM were also performed. *P *value of < 0.05 indicates significant.

## Results

### Clinical and biochemical characteristics of the study subjects

**Figure 1:**
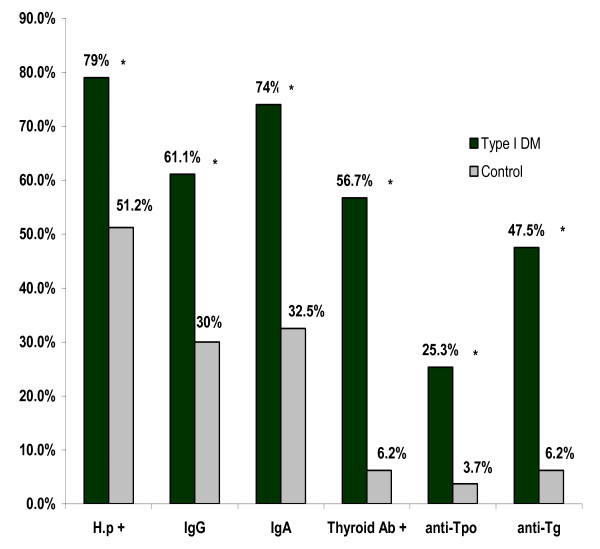
Seoprevalence of anti-H. pylori and anti-thyroid antibodies in patients with type 1 diabetes and healthy controls, * *P *is significant if < 0.05.

Seroprevalence of H. pylori was significantly higher in patients with T1DM than in healthy controls [79% (128/162) vs. 51.2% (41/80), p < 0.001]. Anti- H. pylori IgG was positive in 61.1% (99/162) of patients with T1DM and 30% (24/80) of controls, p < 0.001, anti-H. pylori IgA was positive in 74% (120/162) of patients with T1DM and 32.5% (26/80) of controls, p < 0.001 [Figure 1]. There was no statistic significant difference in the prevalence of dyspeptic symptoms between patients with T1DM and healthy controls (4.3% vs. 3.7%, p = 0.83). The mean ± SD of HbA1c levels during the study period were 8.2 ± 1.75 [Table 1].

Thyroid autoimmunity was significantly higher in patients with T1DM than in healthy controls [56.7% (92/162) vs. 6.2% (5/80), p < 0.001]. Anti-TPO was positive in 25.3% (41/162) of patients with T1DM and 3.7% (3/80) of controls, p < 0.001, anti-Tg was positive in 47.5% (77/162) of patients and 6.2% (5/80) of controls, p < 0.001 [Figure 1].

### Comparison between H. pylori positive and H. pylori negative patients with T1DM

Patients with T1DM were divided, according to H. pylori status, into 2 groups: H. pylori positive and H. pylori negative. The 2 groups were compared for age, duration of diabetes, thyroid autoimmunity, anti-TPO and anti-Tg autoantibodies. There was no significant difference in age between H. pylori positive and negative patients with T1DM. The mean T1DM duration and HbA1c were significantly greater in H. pylori positive than in H. pylori negative patients with T1DM (8.9 ± 8.6 vs. 4.22 ± 2.35, p < 0.001; 8.3 ± 1.58 vs. 6.8 ± 2.3, p < 0.001 respectively). Thyroid autoimmunity (63.2% vs. 32.3%, OR 3.6, 95% CI: 1.5- 8.7, p = 0.002), anti-TPO (28.9% vs. 11.7%, OR 3.05, 95% CI: 0.93-11.1, p = 0.04) and anti-Tg (53.1% vs. 26.4%, OR 4.7, 95% CI: 1.87-12.2, p < 0.001) were significantly higher in H. pylori positive compared to H. pylori negative patients with T1DM [Table 2]. With respect to sex, anti-TPO and anti-Tg antibodies (38.3% vs. 12.7%, OR 4.27, 95% CI: 1.57-11.9, p = 0.002; 68.4.9% vs 32.7%, OR 4.47, 95% CI: 1.9-10.17, p < 0.001 respectively) were higher in female H. pylori positive patients with TIDM than in males.

**Table 2:** Clinical and biochemical parameters in H pylori positive and H pylori negative patients with T1DM

Parameters	H.P positive (n = 128)	H.P negative (n = 34)	OR	95% CI	*P*- value
Age (years)	20.1 ± 4.6	19.8 ± 4.34	-	-	0.72

Duration of diabetes (years)	8.9 ± 8.6	4.22 ± 2.35	-	-	< 0.001*

HbA1c (%)	8.3 ± 1.58	6.8 ± 2.3			< 0.001*

Thyroid autoimmunity (+)	63.2% (81/128)	32.3% (11/34)	3.6	1.5:8.7	0.002*

Anti-TPO (+)	28.9% (37/128)	11.7% (4/34)	3.05	0.93: 11.1	0.04*

Anti-Tg (+)	53.1% (68/128)	26.4% (9/34)	4.7	1.87: 12.2	< 0.001*

Data are expressed as mean ± standard deviation, numbers or percentages. H.P; helicobacter pylori, OR; odds ratio, CI; confidence interval, HbA1c; glycated hemoglobin, Anti-TPO; anti-thyroid peroxidase, Anti-Tg; anti-thyroglobulin, * *P *is significant if < 0.05.

### Comparison between thyroid autoimmunity positive and thyroid autoimmunity negative patients with T1DM

Patients with T1DM were divided, according to thyroid autoimmunity status, into 2 groups: thyroid autoimmunity positive and thyroid autoimmunity negative. The 2 groups were compared for age, duration of diabetes, H. pylori seopositivity, anti-H. pylori IgG and IgA antibodies. With regard to age, duration of diabetes and HbA1c, there were no statistic significant differences between thyroid autoimmunity positive and thyroid autoimmunity negative groups. In patients with positive thyroid autoimmunity as compared to autoimmunity negative, significantly higher H. pylori seopositivity (92.3% vs. 61.4%, OR 7.6, 95% CI: 2.8-12.6, p < 0.001), anti-H. pylori IgG (70.6% vs. 48.5%, OR 2.5, 95% CI: 1.27-5.15 p = 0.007) and IgA (86.9% vs. 57.1%, OR 5, 95% CI: 2.18-11.6, p < 0.001) were found [Table 3]. With respect to sex, only IgG (82.5% vs. 44.8%, OR 5.8, 95% CI: 1.97-17.5, p < 0.001) was higher in female patients with thyroid autoimmunity than in males.

**Table 3:** Clinical and biochemical parameters in thyroid autoimmunity positive and thyroid autoimmunity negative patients with T1DM

Parameters	Thyroid autoimmunity positive (n = 92)	Thyroid autoimmunity negative(n = 70)	OR	95% CI	*P*- value
Age (years)	19.37 ± 2.8	19.32 ± 2.45	-	-	0.92

Duration of diabetes (years)	7.37 ± 2.87	7.12 ± 2.79	-	-	0.54

HbA1c (%)	8.2 ± 1.7	7.85 ± 1.95			0.15

H. pylori positive(+)	92.3% (85/92)	61.4% (43/70)	7.6	2.8: 12.6	< 0.001*

Anti- HP IgG (+)	70.6% (65/92)	48.5% (34/70)	2.55	1.27:5.15	0.007*

Anti- HP IgA (+)	86.9% (80/92)	57.1% (40/70)	5	2.18:11.6	0.001*

Data are expressed as mean ± standard deviation, numbers or percentages. OR; odds ratio, CI; confidence interval, HbA1c; glycated hemoglobin, Anti- H.P IgG; anti-helicobacter pylori IgG, Anti- H.P IgA; anti-helicobacter pylori IgA, * *P *is significant if < 0.05.

### Simple correlation and multiple regression analysis of anti-H. pylori IgG and IgA antibodies titers with other parameters in patients with T1DM

**Figure 2:**
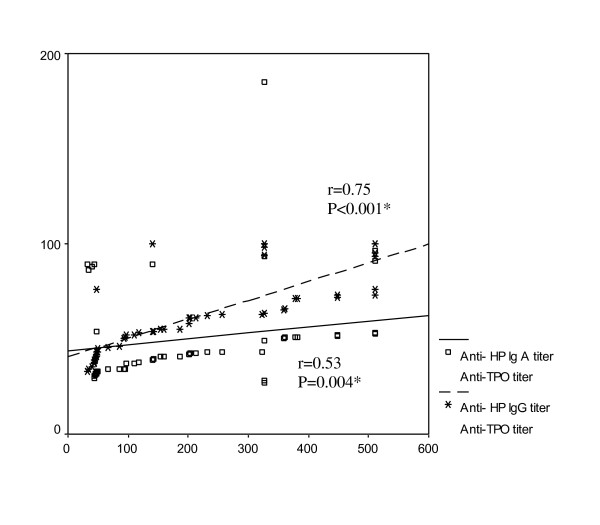
correlations of anti-H. pylori IgG and IgA titers with anti-TPO titer, * *P *is significant if < 0.05.

**Figure 3:**
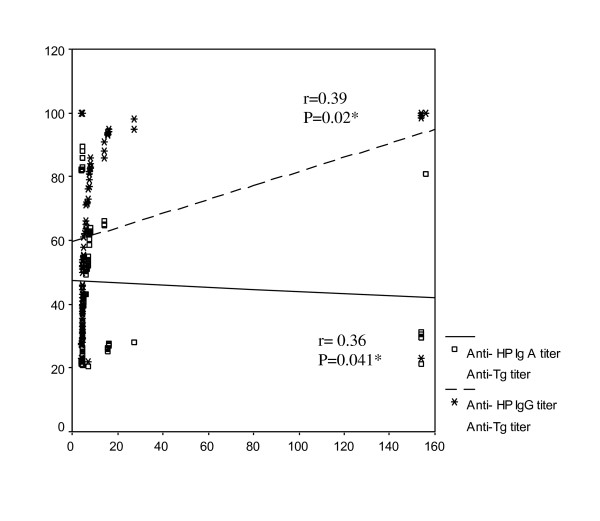
correlations of anti-HP IgG and IgA titers with anti-Tg titer, * *P *is significant if < 0.05.

Anti-H pylori IgG and IgA titers were insignificantly correlated with diabetes duration (r = 0.05, p = 0.85; r = 0.07, p = 0.79 respectively) and HbA1c (r = 0.21, p = 0.35; r = 0.19, p = 0.41 respectively). Anti-H. pylori IgG and IgA titers were positively correlated with anti-TPO (r = 0.75, p < 0.001; r = 0.53, p < 0.004 respectively) [Figure 2] and anti-Tg autoantibodies titers (r = 0.39, p = 0.02; r = 0.36, p = 0.041 respectively) [Figure 3].

With multiple regression analysis anti-TPO and anti-Tg autoantibodies titers remained independently correlated with anti-H. pylori IgG and IgA titers [Table 4].

**Table 4:** Multiple regression analysis with Anti-HP IgG and Anti-HP IgA titers as the dependent variables and Anti-TPO and Anti-Tg titers as the independent variables in patients with T1DM

Antibodies titers	Anti- HP IgG		Anti- HP IgA	
	**B**	***P*-value**	**B**	***P*-value**

Anti-TPO	0.63	< 0.001***	0.54	< 0.001***
Anti-Tg	0.39	< 0.001***	0.245	0.002**

Anti- H.P IgG; anti-helicobacter pylori IgG, Anti- H.P IgA; anti-helicobacter pylori IgA, Anti-TPO; anti-thyroid peroxidase, Anti-Tg; anti-thyroglobulin, * *P *< 0.05** p < 0.01 *** p < 0.001.

## Discussion

The current study shows a higher seroprevalence of H. pylori infection in Egyptian patients with T1DM when compared with control participants, matched for age, sex and socioeconomic status. H. pylori infection was asymptomatic and was significantly associated with diabetes duration and poor glycemic control.

Our results are consistent with previous reports of high prevalence of H. pylori infection in young patients with T1DM [7,13-15] and are in contrast with others which did not demonstrate any difference between the prevalence of H. pylori in patients with diabetes and healthy controls [16,17].

We found no significant difference in the dyspeptic symptoms between patients and healthy controls. The relationship between gastrointestinal symptoms in diabetes and H. pylori infection is controversial. According to some studies, there is no difference between diabetics and non-diabetics concerning the prevalence of H. pylori-related gastroduodenal disorders [18]. On the other hand, some data demonstrated a higher prevalence of H. pylori infection in diabetic patients with dyspepsia as compared with non-diabetics [19].

In agreement with other reports [7,19] this study shows that H. pylori seropositivity was significantly associated with the duration of diabetes. Moreover, HbA1c levels were significantly greater in H. pylori positive patients with T1DM than in H. pylori negative. This is in line with Toporowska-Kowalska et al. [14] and disagrees with Candelli et al. [20]. The poor glycemic control in H. pylori infected patients could be attributed to the increased production of pro-inflammatory cytokines [21] induced by H. pylori gastric infection itself. On the other hand, alteration in glucose metabolism may promote H. pylori colonization [22].

The high prevalence of H. pylori infection in patients with diabetes is generally explained by reduced gastric motility and peristaltic activity which may promote H. pylori colonization [22], various chemical changes in gastric mucosa following non-enzymatic glycosylation of mucins or increased sialic acid [23] which may be involved as a receptor for H. pylori on the cell surface by promoting adhesion of H. pylori to gastric mucosa cells [24] and an impaired non-specific immunity observed in patients with diabetes [25].

In the present study, thyroid autoimmunity was significantly higher in patients with T1DM than in healthy controls; the prevalence rates of anti-TPO and anti-Tg antibodies in T1DM patients of our study were 25.3% and 47.5% respectively.

These results are in accordance with previous reports [9,10,26]. Very recently, Ghawil et al. [27] documented that 23.4% of Libyan TIDM patients had positive anti-TPO antibodies and 7% had positive anti-Tg antibodies. In Iran, Sharifi et al. [28] found that 39.6% of TIDM patients had positive anti-TPO antibodies and 30% had positive anti-Tg antibodies.

Of interest we noted an association between H. pylori and anti-thyroid antibodies; anti-TPO and anti-Tg autoantibodies were significantly elevated in the group of H. pylori seropositive patients with T1DM. On the other hand, patients with T1DM with laboratory signs of thyroid autoimmunity had elevated anti-H. pylori IgG and IgA. In addition, anti-H. pylori IgG and IgA titers were significantly and independently correlated with anti-TPO and anti-Tg titers; the strong positive relations between these antibodies titers suggest that H. pylori infection might be involved in the development of AT in young patients with T1DM.

Our results are consistent with most previous studies [8,29-31] reporting a connection between H. pylori infection and autoimmune thyroiditis. Larizza et al. [32] found a significant interaction between HLA-DRB1*0301, a well-known marker of autoimmunity, and H. pylori infection in patients with AT and not controls. This allele has been specifically involved in both cellular and humoral reactions against self structures such as thyrocytes [33]. Furthermore, our results are parallel those of Triantafillidis et al. [34], who found a relationship between H. pylori infection and the presence of high titers of anti-TPO and anti-Tg autoantibodies. Decrease of antithyroid autoantibodies titers has been observed in patients with AT after eradication of H pylori infection [29].

On the contrary, other studies showed no differences in the serum levels of thyroid autoantibodies in patients with and without H. pylori infection [35,36]. Moreover, Franceschi et al. [35] found a similar prevalence of H. pylori infection in patients with Hashimoto's thyroiditis and controls.

In our patients' group we found no significant association between thyroid autoimmunity and age or the duration of diabetes. The highest prevalence of thyroid antibodies in patients with T1DM was observed after the age of 15 years [37-39]. It is known that the maximum autoimmune activity is observed during puberty [40]. In agreement with our results, Kordonouri et al. [38] found insignificant role of diabetes duration in the development of thyroid autoimmunity.

We have noticed a significant association between the thyroid autoimmunity with female sex in H. pylori positive patients with T1DM. This is Consistent with De Block et al. [37] who reported a 3-fold risk of anti-TPO antibody positivity in female adolescents and young adults with diabetes in comparison with males. Similar findings were reported by Kordonouri et al. [26,38]. In patients with T1DM [41] oestradiol seemed to accelerate the progression of autoimmune diseases via enhancing the pathway of T helper type 2 cells, while androgens had a protective effect [40].

The etiologic causes for autoimmune thyroiditis development are multifactorial, involving genetic predisposition [33,42] and external factors, most common being infections such as H. pylori [8]. The putative mechanism to explain how H. pylori infection in the stomach can pathogenically influence remote organs is the induction of an autoimmune reaction by cross reactivity with thyroid antigens [43]. Moreover, H. pylori infection induces an acute polymorphonuclear infiltration in the gastric mucosa. If the infection is not effectively cleared, this acute cellular infiltrate is gradually replaced by an immunologically-mediated, chronic, predominantly mononuclear cellular infiltrate [44]. The latter is characterized by the local production and systemic diffusion of pro-inflammatory cytokines [21], which may exert their effect in remote tissues and organs [45]. Therefore, a pathogenic link between H. pylori infection and diseases characterized by activation of inflammatory mediators and or induction of autoimmunity might exist [46].

## Conclusion

results of this study support the idea of a connection between H pylori infection and the occurrence of anti-TPO, anti-Tg autoantibodies and AT in young patients with T1DM, so helicobacter pylori infection could be considered as an environmental trigger for development of AT in T1DM. Young patients with T1DM should be screened for H. pylori infection.

## List of abbreviations

H pylori: helicobacter pylori; T1DM: type 1 diabetes mellitus; AT: autoimmune thyroiditis; anti-Tg: anti-thyroglobulin; anti-TPO: anti-thyroid peroxidase; FT4: Free-thyroxine; TSH: thyroid stimulating hormone; HbA1c: glycated hemoglobin.

## Competing interests

The authors declare that they have no competing interests.

## Authors' contributions

MME drafted the manuscript, conceived the study, and participated in its design and coordination. AKE helped to draft the manuscript and participated in the coordination of the study. AAE and SAA carried out the laboratory studies. All authors read and approved the final manuscript.
